# Association between TUG and Anthropometric Values, Vibration Perception Threshold, FHSQ and 15-D in Type 2 Diabetes Mellitus Patients

**DOI:** 10.3390/ijerph17062018

**Published:** 2020-03-19

**Authors:** Francisco Javier Domínguez-Muñoz, José Carmelo Adsuar, Jorge Carlos-Vivas, Santos Villafaina, Miguel Angel Garcia-Gordillo, Miguel Ángel Hernández-Mocholi, Daniel Collado-Mateo, Narcis Gusi

**Affiliations:** 1 Physical Activity and Quality of Life Research Group (AFYCAV), Faculty of Sport Science, University of Extremadura, 10003 Cáceres, Spain; fjdominguez@unex.es (F.J.D.-M.); svillafaina@unex.es (S.V.); mhmocholi@unex.es (M.Á.H.-M.); ngusi@unex.es (N.G.); 2 Health Economy Motricity and Education (HEME), Faculty of Sport Science, University of Extremadura, 10003 Cáceres, Spain; jadssal@unex.es (J.C.A.); jorge.carlosvivas@gmail.com (J.C.-V.); 3 Facultad de Administración y Negocios, Universidad Autónoma de Chile, sede Talca 3467987, Chile; 4 Centre for Sport Studies, Rey Juan Carlos University, 28943 Fuenlabrada, Madrid, Spain; danicolladom@gmail.com

**Keywords:** diabetes mellitus, TUG, FHSQ, HRQoL, 15D, vibration perception threshold

## Abstract

*Background*: Diabetes Mellitus (DM) is a chronic disease and it is characterized by reduced insulin sensitivity and/or impaired insulin production. It affects approximately 415 million people worldwide and involves a variety of complications. DM has a number of complications, including diabetic neuropathy. All of these complications can have effects on body composition, vibration perception threshold (VPT), foot health and health-related quality of life (HRQoL). *Objective*: The aim of this study is to determine the correlation between the Timed Up and Go (TUG), VPT, Foot Health Status Questionnaire and 15-D Questionnaire in type 2 diabetes mellitus (T2DM) patients. *Methodology*: A total of 90 T2DM patients (56 men and 34 women) were evaluated on their body composition, VPT, the foot health status through the FHSQ, the HRQoL was evaluated through the 15-D Questionnaire and the TUG test was performed. *Results*: Statistically significant associations were found between TUG and lean and fat mass, VPT, the sections “General Foot Health” and “Physical Activity” in the FHSQ questionnaire, and the 15D total score and its sections “Mobility” and “Depression”. *Conclusions*: There is a moderate direct correlation between the Timed Up and Go and the fat mass percentage and the vibration perception threshold. Moreover, there is a moderate inverse correlation between Timed Up and Go and fat-free mass, foot health and health-related quality of life in T2DM patients. Therefore, Timed Up and Go can be a tool to assist clinicians in monitoring and managing T2DM patients.

## 1. Introduction

Type-2 Diabetes Mellitus (T2DM) is a chronic disease characterized by chronic hyperglycemia derived from either impaired insulin secretion or impaired insulin action or both [1]. According to epidemiological studies, diabetes mellitus (DM) affects 415 million people worldwide [2], but the prevalence may be higher since, according to the International Diabetes Federation, 46.5% of DM patients have not yet been diagnosed.

DM complications, such as diabetic neuropathy [3], may affect health-related quality of life (HRQoL). One instrument to assess the HRQoL in T2DM patients [4,5] is the 15-D questionnaire, which consists of 15 sections. In addition, it is important to know the foot health status of patients, that may be measured using different tools. One of these tools is the Foot Health Status Questionnaire (FHSQ). The FHSQ is a valid and reliable instrument related to HRQoL, specific to the foot, which was initially developed to assess the surgical treatment results of common foot diseases [6]. This questionnaire has been previously used in DM patients or those who have DM-related ulcers [7,8]. One of the causes that can worsen the foot health state is the loss of sensitivity to vibration, which is related to diabetic peripheral neuropathy.

Diabetic peripheral neuropathy is characterized by a progressive loss of sensitivity in the most distal body parts, even affecting nociceptive skin small-diameter fibers [9]. This affectation can even reach the neuromotor fibers, leading to muscle weakness. In this regard, T2DM patients usually present a 17% and 14% reduction in knee flexor and extensor muscle strength, respectively [10]. It is important to consider these aspects, since this type of DM complication may affect balance and cause posture and gait alterations [11], and; consequently, it can affect the foot and ankle proprioception [12] as well as feet sensitivity [13]. Moreover, this foot sensitivity loss is associated with an increased risk of foot ulcers [14]. In this regard, there are different tools to examine the degree of impairment in foot sensitivity loss and to evaluate the vibration perception threshold (VPT). Different instruments have been used to define VPT. Particularly, the Vibratron II biotensiometer has reported good reliability in different populations [15,16,17]. Previous studies have shown the relationship between VPT and risk of falling [18], walking speed [19] and mobility disability [20]. Another test that can assess agility and balance is the Timed Up and Go (TUG). The reliability of this useful test (it can be administered in a very short time and it does not require specialized equipment) has been previously evaluated in T2DM patients and it was also related to balance [21]. Thus, this test can be used in primary care centers and in this sense, this article could be the first step to explore the possibilities offered by the TUG as a relevant test in a first screening of a high sensitivity threshold, of poor foot health or a decrease in health-related quality of life.

Based on the above, it seems important to know if there is a correlation between TUG values, VPT, foot health using the FHSQ questionnaire and HRQoL, through the 15-D questionnaire in T2DM patients. To our knowledge, to date, there is no study that has evaluated the relationship between these instruments. Therefore, the aim of this study was to determine the possible relationship between TUG and 3 variables: (1) VPT, (2) FHSQ questionnaire and 15-D questionnaire in T2DM patients.

## 2. Materials and Methods

### 2.1. Study Design

A descriptive cross-sectional design was conducted in order to analyze the relationship between the Timed Up and Go test (TUG) and anthropometric characteristics, the Foot Health Status Questionnaire (FHSQ), vibration perception threshold (VPT) and health-related quality of life (HRQOL) in T2DM patients. The study was approved by the Bioethics and Biosafety Committee at the University of Extremadura (44/2012), according to the Declaration of Helsinki ethical standards and the National legislation on bioethics, biomedical research and sample confidentiality. All participants were informed about the procedures and signed an informed consent form prior to starting the study.

### 2.2. Sample Size Estimation

Sample size computations were estimated, assuming alpha and beta risks of 0.05 and 0.20, respectively, in bilateral contrast. Results revealed that at least 85 T2DM patients were needed, accepting a 0.30 correlation coefficient. This correlation coefficient was selected assuming that it means a moderate correlation according to Cohen’s classification [22].

### 2.3. Participants

Ninety participants diagnosed with T2DM (34 females and 56 males), from the “Manuel Encinas” Health Centre in Cáceres (Extremadura, Spain) were recruited for this study. Participants would have to fulfill a set of criteria to be eligible: (a) men or women diagnosed with T2DM, (b) aged between 40 and 85 years old, and (c) have read, accepted and signed the written informed consent.

### 2.4. Procedures and Assessments

#### 2.4.1. Initial Questionnaire

Participants were asked about their age and the year when T2DM was diagnosed.

#### 2.4.2. Diabetes Status Measure

The glycosylated hemoglobin (HbA1c) was assessed through a blood analysis in order to check the patients’ status. This test is usually used to make a diagnosis or check the diabetes management. The thresholds were: <5.7% normal; 5.7–6.4% prediabetes; and >6.5% T2DM.

#### 2.4.3. Timed Up and Go

Participants start seated on a standard chair with a height close to 46 cm, with their back against the seat-back. At the “go” signal, patients should get up, walk a 3-m distance, turn around a cone and return to the chair to sit again. They were asked to walk as fast as possible in a comfortable and safe way. A stopwatch was used to measure the time used to complete the test, in seconds. Prior to the outcomes’ registration trial, all participants performed a familiarization attempt [23]. This instrument previously showed excellent reliability in patients with T2DM [21]. This test was validated to predict the likelihood of falls in older adults living in the community [24].

#### 2.4.4. Anthropometric Measures

Bodyweight and composition were evaluated using a Tanita BC-418 bioimpedancemeter and height was measured with a stadiometer Seca. Additionally, body mass index (BMI) was calculated through the equation: BMI = weight [kg]/(height [m]^2^).

#### 2.4.5. Foot Health Status Questionnaire (FHSQ)

Foot health status was evaluated through the Foot Health Status Questionnaire (FHSQ). This questionnaire consists of three different sections. The first two sections reference the 8 dimensions that compose the questionnaire, while the third collects socio-demographic data. Specifically, four dimensions were extracted from the first section, evaluated through 13 questions: (1) foot pain, (2) foot function, (3) footwear and (4) general foot health. The second section refers to another 4 dimensions, evaluated through 20 questions: (1) general health, (2) physical activity, (3) social capacity and (4) vigor. These 33 questions used to calculate the 8 dimensions are answered using a Likert scale from 1 to 5. Each dimension is scored between 0–100, where 0 is the worst possible foot health status and 100 is the best possible foot health status. Both the original version and the translated Spanish version of this questionnaire have proven to be valid and reliable [25,26]. This questionnaire has been validated in different podiatric diseases, such as skin, neurological and musculoskeletal diseases, and it has also been used to determine the effectiveness of foot orthoses [27,28].

#### 2.4.6. Vibration Perception Threshold (VPT)

The VPT was evaluated using the Vibraton II device (Sensortek, In. Clifton, NJ, USA), which consists of a vibration controller and two vibration modules (one for each side). Data were collected using a standardized procedure provided by the manufacturer, which have been also used in several previous studies [15,29]. Once data were taken, the alpha-trimmed average of the last 5 failures and the last 5 hits were taken, without considering the highest error or the lowest hit. Similar to the measurement procedure, the computations of VPT were performed following the manufacturer’s guidelines, which have also been used in previous studies such as Deng et al. [15,29] The Vibraton II measures in vibration units, which are related to the amplitude of movement in microns, following the equation: A = x^2^/2 (where x is the vibration unit [vu] and A is the amplitude in microns [μ]). The scoring goes from 0–20, where 0 is the best VPT and 20 is the worst possible VPT. Validation is difficult in the absence of a gold standard, so it was indirectly evaluated by studying the relationship between nerve conduction rates and vibration thresholds. In this regard, a high correlation has been found between nerve conduction rates and vibration thresholds [30]. Asymptomatic diabetic patients have significantly higher thresholds than non-diabetic individuals, suggesting that this measure may be useful in detecting subclinical neuropathy [31].

#### 2.4.7. Health-Related Quality of Life (HRQoL)

Health-related quality of life was assessed using the 15-D questionnaire [32], which consists of 15 dimensions: mobility, vision, hearing, breathing, sleeping, eating, speech, excretion, usual activities, mental function, discomfort and symptoms, depression, distress, vitality, and sexual activity. Each dimension has five possible answers from less to more impaired. The total score represents the person’s health status (1—full HRQoL, 0—death). This questionnaire has been previously used in DM patients in several countries [33,34], including Spain [35]. Content and construct validity was done where the questionnaire 15-D was correlated with EQ-5D, NHP and SF-20 [32].

### 2.5. Statistical Analytics

Statistical analyses were performed using the software SPSS 21 for Windows (SPSS In., Chicago, IL, USA). Data are presented as mean and standard deviation. The *Kolmogorov–Smirnov* test was performed to explore the distribution of the data. Since data did not follow a normal distribution, the Spearman correlation coefficient was used to establish the degree of correlation between TUG and the other variables. Moreover, since it is multiple correlations, Bonferroni post hoc was applied to reduce the probability of a type I error bias, establishing the level of significance at *p* ≤ 0.001. To interpret the correlation coefficient, Cohen’s classification thresholds have been followed [22]: 0.30 to 0.59 moderate; 0.6 to 0.79 high and ≥ 0.8 excellent.

## 3. Results

Table 1 shows the studied sample characteristics (*n* = 90).

Table 2 shows Spearman’s correlation coefficients between TUG and anthropometric data. There is a moderate direct correlation between TUG and fat mass percentage. Also, TUG correlates moderately but inversely with total body water and fat-free mass.

Table 3 reports Spearman’s correlation coefficient that relates TUG to VPT and FHSQ. The outcomes indicate that there is a moderate direct correlation between TUG and VPT. Additionally, physical activity, general foot health and vigor dimensions correlate moderately and inversely with TUG.

Table 4 shows Spearman’s correlation coefficient between TUG and health-related quality of life from the 15-D questionnaire and the 15 dimensions that conform it. There is a moderate inverse correlation between TUG and HRQoL, as well as between TUG and mobility, discomfort and symptoms and depression 15-D questionnaire dimensions.

## 4. Discussion

The main finding of this study is the significant association between TUG and anthropometric data, the Foot Health Status Questionnaire FHSQ, the VPT and the 15-D HRQoL questionnaire. To our knowledge, this is the first study that relates TUG to anthropometrics values, the FHSQ, the VPT and 15-D questionnaires in T2DM.

In line with our results, a previous study by Mohd Said, Manaf, Adli Bukry, Justine [36] analyzed the correlation between TUG and anthropometric data in older people without T2DM. Additionally, they studied the relationship by type of footprint and reported significant correlations between TUG and bodyweight in the group of supinator footprint people. However, they reported no correlation comparing TUG with the rest of the subgroups and the height. This is in contrast to our study, where results show that TUG correlates with height, fat mass percentage, total body water, fat-free mass and BMI. These results are in line with a previous study by Brady, Straight, Schmidt, Evans [37] which reported that people with a higher rate of obesity walk slower on the 8-Foot Up-and-Go test. Moreover, our results are also in line with a previous study [38], where they reported that increasing age and high values of obesity have an impact on mobility-related problems.

As far as the association between TUG and health-related quality of life is concerned, there is, to our knowledge, no study to date that has studied this relationship in T2DM patients. In this regard, this is the first study to explore the association between TUG and health-related quality of life, as measured by 15-D in T2DM patients. Results showed that TUG inversely correlated with the total score of the 15-D questionnaire and with three of its dimensions (depression, discomfort and symptoms, and mobility). These results are in line with previous studies in which TUG was correlated with other quality of life questionnaires [39,40,41]. In Parkinson, TUG correlated with Parkinson’s Disease Questionnaire—39 items [39]. In older people with the Korean form of Geriatric Depression Scale (K-GDS) [40] and Short Form-12 (SF-12) [41], there were statistically significant associations of TUG with depression assessed with K-GDS, and with the physical component and general health assessed with SF-12.

Regarding the association between TUG and VPT, there is a previous study in which a moderate direct correlation has been found between TUG and VPT in people with multiple sclerosis [42]. However, to our knowledge, this is the first study that correlates TUG with VPT in T2DM patients. Two studies have also investigated the possible relationship between USVP and TUG in older people [43] and people with spinal canal stenosis [44]. However, no statistically significant correlations were found in any of them.

To our knowledge, this is the first study that correlates TUG with foot health status across the eight dimensions of the FHSQ questionnaire. The TUG inversely and moderately correlated with the “general foot health”, “physical activity” and “vigor” dimensions of the FHSQ questionnaire. The inverse relationship between TUG and physical activity level has been previously found in previous studies, focused in other populations [45,46]. However, to our knowledge, the relationship between TUG and “general foot health” and “vigor” had not been previously studied.

### 4.1. Clinical Implications

If the relationships found here between TUG and the other variables evaluated are confirmed, physicians evaluating patients with type 2 diabetes may use TUG as an initial screening for possible loss of peripheral sensation, poor foot health, or poor health-related quality of life. Obviously, this would be a complementary test, given the multifactorial nature of the threshold for sensitivity to vibration, poor foot health or health-related quality of life, making it necessary to refer patients to a specialist medical service to determine whether they actually have any of these problems. The TUG would be a useful test to be used as an initial screening for other medical problems, since it is easy and quick to perform and does not require specialized equipment.

### 4.2. Limitations

The current study has some limitations that should be taken into account. In future studies, the sample of men and women should be expanded, to have sufficient statistical power to be able to disaggregate data by sex. Some authors consider Bonferroni’s adjustment too conservative and propose the use of other alternatives for more efficient control of type 1 error [47].

## 5. Conclusions

Statistically significant associations between Timed Up-and Go and body composition, vibration perception threshold, foot health status and health-related quality of life were found in T2DM patients.

## Figures and Tables

**Table 1:** Sample characteristics (*n* = 90).

Variables	Mean	SD	Median	IQR
Age (years)	65.64	8.65	66.00	9.00
HbA1c (%)	6.78	1.02	6.55	1.30
Weight (kg)	80.63	16.19	77.60	18.05
Height (cm)	164.89	10.00	165.50	13.00
BMI (kg/m^2^)	29.65	4.39	28.65	4.75
Fat Mass Percentage (%)	32.97	7.49	31.99	12.07
Total Body Water (%)	49.08	5.47	49.80	8.72
Fat-Free Mass (%)	66.84	8.08	68.00	12.13
Basal Metabolic Rate (kcal)	1631.61	482.97	1526.00	474.00
Years from Diagnostic (years)	9.96	8.83	7.00	12.00
Timed Up and Go (s)	8.12	2.01	7.69	1.88
VPT (vu)	5.66	2.55	5.60	4.16
Foot Health Status Questionnaire (FHSQ)				
Foot Pain	88.77	20.58	100.00	21.25
Foot Function	94.58	16.07	100.00	0.00
Footwear	76.57	38.03	100.00	33.34
General Foot Health	57.36	24.85	60.00	30.00
General Health	66.22	22.91	70.00	40.00
Physical Activity	80.61	21.81	88.88	22.22
Social Capacity	91.52	20.42	100.00	0.00
Vigor	67.13	23.96	68.75	37.50
15-D Quality of Life Questionnaire (15-D)				
Mobility	0.93	0.16	1.00	0.00
Vision	0.91	0.17	1.00	0.22
Hearing	0.88	0.19	1.00	0.26
Breathing	0.89	0.18	1.00	0.31
Sleeping	0.75	0.32	1.00	0.70
Eating	1.00	0.00	1.00	0.00
Speech	0.98	0.06	1.00	0.00
Elimination	0.89	0.20	1.00	0.00
Usual Activities	0.96	0.10	1.00	0.00
Mental Function	0.82	0.21	1.00	0.36
Discomfort and Symptoms	0.88	0.20	1.00	0.30
Depression	0.88	0.21	1.00	0.24
Distress	0.86	0.24	1.00	0.28
Vitality	0.89	0.18	1.00	0.23
Sexual Activity	0.72	0.34	1.00	0.56
15-D Total Score	0.89	0.09	0.91	0.10

SD: Standard deviation; IQR: Interquartile range; HbA1c: Glycosylated Hemoglobin; BMI: Body mass index; VPT: vibration perception threshold; vu: vibration units; kg: kilograms; s: seconds; Kcal: kilocalories. FHSQ questionnaire scores range from 0 to 100 and 15-D questionnaire scores vary between 0 and 1.

**Table 2:** Correlation between Timed Up and Go test and Anthropometric data in type 2 diabetes mellitus patients (*n* = 90).

Body Composition Variables	Timed Up and Go	
	Spearman’s Rho	*p* *
Weight (kg)	0.069	0.518
Height (cm)	−0.233	0.027
BMI (kg/m^2^)	0.251	0.017
Fat Mass Percentage (%)	0.399	<0.001
Total Body Water (%)	−0.401	<0.001
Fat-Free Mass (%)	−0.440	<0.001
Basal Metabolic Rate (Kcal)	0.000	0.997

Kg: kilograms; cm: centimeters; m: meters; Kcal: kilocalories. * *p* refers to the p-value of Spearman’s correlation coefficient.

**Table 3:** Correlation between Timed Up and Go test, vibration perception threshold (VPT) and Foot Health Status Questionnaire (FHSQ) Dimensions in type 2 diabetes mellitus population (n = 90).

Variables	Timed Up and Go	
	Spearman’s Rho	*p* *
VPT (vu)	0.351	0.001
Foot Health Status Questionnaire (FHSQ)		
Foot Pain	−0.298	0.004
Foot Function	−0.280	0.008
Footwear	−0.169	0.111
General Foot Health	−0.477	<0.001
General Health	−0.085	0.425
Physical Activity	−0.362	<0.001
Social Capacity	−0.272	0.009
Vigor	−0.340	0.001

VPT: vibration perception threshold; vu: vibration units. * *p* refers to the *p*-value of Spearman’s correlation coefficient.

**Table 4:** Correlation between Timed Up and Go test and 15-D Quality of Life Questionnaire Dimensions and total score in type 2 diabetes mellitus population (*n* = 90).

HRQoL Variables	Timed Up and Go	
	Spearman’s Rho	*p* *
15-D Total Score	−0.440	<0.001
15-D Quality of Life Questionnaire (15-D)		
Mobility	−0.383	<0.001
Vision	−0.172	0.105
Hearing	−0.089	0.407
Breathing	−0.244	0.021
Sleeping	−0.297	0.004
Eating	N/A	N/A
Speech	−0.131	0.219
Elimination	−0.231	0.029
Usual Activities	−0.177	0.094
Mental Function	−0.069	0.516
Discomfort and Symptoms	−0.330	0.001
Depression	−0.386	<0.001
Distress	−0.178	0.094
Vitality	−0.285	0.006
Sexual Activity	−0.096	0.366

N/A: Not Applicable. * *p* refers to the *p*-value of Spearman’s correlation coefficient.
